# More Ethics of Controversy

**Published:** 1919-03-01

**Authors:** Isabel Macdonald

**Affiliations:** Secretary, Royal British Nurses' Association.


The Editor of THE HOSPITAL,
29 Southampton Street.

SIR,—I am in receipt of your communication of 18th instant, and note that you refuse to publish the letter† I forwarded in reply to Sir Cooper Perry's.—I am, dear Sir, yours truly,

(Signed) ISABEL MACDONALD,
Secretary,
Royal British Nurses' Association.

† [The letter was published in full in the smallest type in last week's THE HOSPITAL, p. 460.]

